# Supplementary material for: The Small RNA Universe of Capitella teleta
Source: Front Mol Biosci. 2022 Feb 25;9:802814. doi: 10.3389/fmolb.2022.802814 (PMC8915122; doi:10.3389/fmolb.2022.802814)
Supplement: Supplementary file 1 [file DataSheet1.ZIP › Supplement/known/CAPTEscaffold_12304_44299.pdf]

Provisional ID : CAPTEscaffold_12304_44299
Score total : 2286.3
Score for star read(s) : 3.9
Score for read counts : 2280.1
Score for mfe : 1.3
Score for randfold : 1.6
Score for cons. seed : -0.6
Total read count : 4484
Mature read count : 4404
Loop read count : 0
Star read count : 80

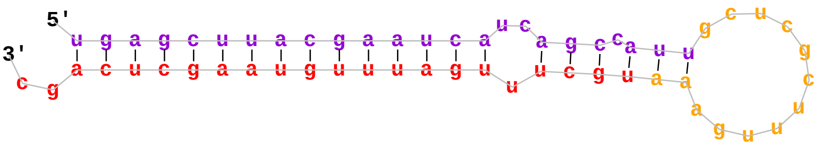

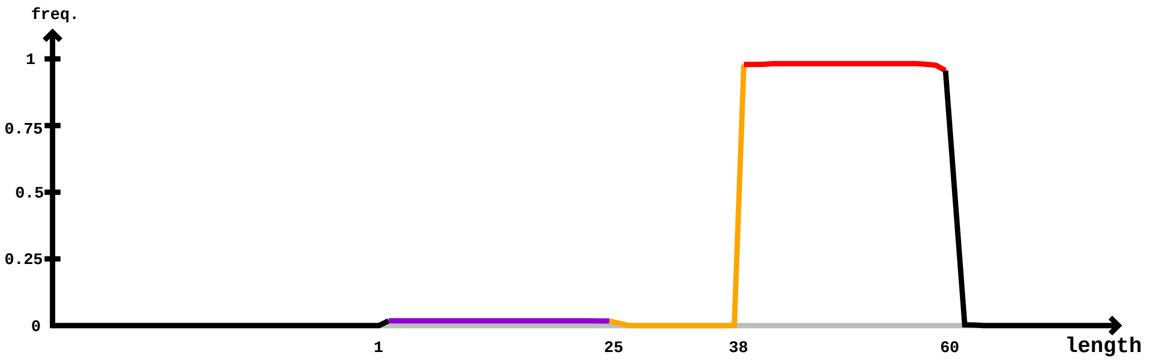

Star Mature

```
5'- caauucagcccagagaagacggaaccagugacuugagcuuacgaaucaucagccauugcucgcuuugaaaugcuuugauuuguaagcucagcaggcgugguuucaaaug   -3'    obs
    caauucagcccagagaagacggaaccagugacuugagcuuacgaaucaucagccauugcucgcuuugaaaugcuuugauuuguaagcucagcaggcgugguuucaaaug          exp
    caauucagcccagagaagacggaaccagugacuugagcuuacgaaucaucagccauugcucgcuuugaaaugcuuugauuuguaagcucagcaggcgugguuucaaaug          known
    ....................((((((((...(((((((((((((((((..(((.(((...........)))))).))))))))))))))))...)).)))))))).....    reads      mm        sample
    .................................ugagcuuacgaaucaucagcca......................................................    3          0         seq
    .................................ugagcuuacgaaucaucagccauu....................................................    74         0         seq
    .................................ugagcuuacgaaucauAagccauu....................................................    1          1         seq
    .................................ugagcuuacAaaucaucagccauu....................................................    1          1         seq
    .................................ugagcuuacgaaucaucagccauuU...................................................    1          1         seq
    .....................................................................Cugcuuugauuuguaagcucagc.................    2          1         seq
    ......................................................................ugcuuugauuuguaagcuc....................    11         0         seq
    ......................................................................ugcuuugauuuguaagcuca...................    16         0         seq
    ......................................................................ugcuuugauuuguaagcucag..................    90         0         seq
    ......................................................................ugcuAugauuuguaagcucagc.................    3          1         seq
    ......................................................................ugcuuugauuuguaagcuUagc.................    4          1         seq
    ......................................................................ugcuuugauuuguaagcucagU.................    32         1         seq
    ......................................................................ugcuuugauuuguaGgcucagc.................    83         1         seq
    ......................................................................ugcuuugGuuuguaagcucagc.................    1          1         seq
    ......................................................................Cgcuuugauuuguaagcucagc.................    2          1         seq
    ......................................................................ugcuuugauuuguaagcucagA.................    47         1         seq
    ......................................................................ugAuuugauuuguaagcucagc.................    1          1         seq
    ......................................................................ugcuuCgauuuguaagcucagc.................    1          1         seq
    ......................................................................ugcuuugauuugCaagcucagc.................    1          1         seq
    ......................................................................ugcuuugUuuuguaagcucagc.................    1          1         seq
    ......................................................................ugcuuuUauuuguaagcucagc.................    2          1         seq
    ......................................................................ugcuuugaAuuguaagcucagc.................    1          1         seq
    ......................................................................Ngcuuugauuuguaagcucagc.................    4          1         seq
    ......................................................................uAcuuugauuuguaagcucagc.................    1          1         seq
    ......................................................................Ggcuuugauuuguaagcucagc.................    1          1         seq
    ......................................................................ugcuuuAauuuguaagcucagc.................    5          1         seq
    ......................................................................ugcuuugauuuguaagcucagc.................    3971       0         seq
    ......................................................................ugcuuugauuuguaagcAcagc.................    1          1         seq
    ......................................................................ugcuuugauAuguaagcucagc.................    1          1         seq
    ......................................................................ugcuuugauuuguaaAcucagc.................    1          1         seq
    ......................................................................Agcuuugauuuguaagcucagc.................    10         1         seq
    ......................................................................ugcuuugauuuguaagAucagc.................    4          1         seq
```

Star **Mature**

```
caauucagcccagagaagacggaaccagugacuugagcuuacgaaucaucagccauugcucgcuuugaaaugcuuugauuuguaagcucagcaggcgugguuucaaaug
......................................................................ugcuuugauuuguaagcuGagc.................  1   1  seq
......................................................................ugcuuugaCuuguaagcucagc.................  1   1  seq
......................................................................ugGuuugauuuguaagcucagc.................  2   1  seq
......................................................................ugcuuugauuuAuaagcucagc.................  1   1  seq
......................................................................ugcuuugauuAguaagcucagc.................  2   1  seq
......................................................................ugcuuugauuuguaagUucagc.................  4   1  seq
......................................................................ugcAuugauuuguaagcucagc.................  2   1  seq
......................................................................ugcuuugauuuguaagcucagca................  2   0  seq
......................................................................ugcuuugauuuguaagcucagcC................  3   1  seq
......................................................................ugcuuugauuuguaagcucagcU................  75  1  seq
........................................................................cuuugauuuguaagcucagc.................  1   0  seq
........................................................................cuuugauuuguaagcucagcag...............  1   0  seq
.........................................................................uuugauuuguaagcucagc.................  1   0  seq
.........................................................................uuugauuuguaagcucagcag...............  2   0  seq
.........................................................................uuugauuuguaagcucagcagU..............  4   1  seq
.........................................................................uuugauuuguaagcucagcagA..............  5   1  seq
```
